# Monitoring arts and psychomotor therapies: Further development and validation of the Self-expression and Emotion-Regulation in Arts and Psychomotor Therapies Scale (SERAPTS)

**DOI:** 10.1371/journal.pone.0336170

**Published:** 2025-11-14

**Authors:** Suzanne Haeyen, Eric Noorthoorn, Evelien Joosten

**Affiliations:** 1 HAN University of Applied Sciences, Research Group Arts & Psychomotor Therapies in Health Care, School of Health Studies, Nijmegen, The Netherlands; 2 GGNet, Centre for Mental Health, Scelta, Expert Centre for Personality Disorders, Warnsveld, The Netherlands; 3 Radboud university Nijmegen, Department of Clinical psychology, Nijmegen, The Netherlands; Amity University, INDIA

## Abstract

**Background:**

The present study is an extension of previous research, which resulted in the development of a valid, reliable and user-friendly tool for monitoring art therapy. The purpose of this study was to examine the extended reliability and sensitivity to change of the adapted version of the above scale, now called the Self-expression and Emotion Regulation in Arts and Psychomotor Therapies Scale (SERAPTS) for art, dance, drama, music and psychomotor therapy.

**Methods:**

A pre-post design study was conducted on adult patients diagnosed with emotion regulation problems or personality disorder(s) cluster B/C. The study’s sample size was determined by the researchers to be 96 participants for the purpose of testing internal consistency, and 67 participants for the purpose of testing sensitivity for change.

**Results:**

An excellent internal consistency was found for all domains of therapy combined (Cronbach’s *α = *0.95). Additionally, high to excellent internal consistencies were identified for dance (*α = *0.88), drama (*α = *0.89), music (*α = *0.95), and psychomotor therapy (*α = *0.95), when considered separately. Furthermore, a significant sensitivity for change was identified within a 3-month timeframe (t = −4.39, p < .001), accompanied by a medium effect size (Cohen’s *d = *0.54).

**Discussion:**

The validity of the tool has been established in previous research. The present study sought to establish the applicability and reliability/sensitivity to change of the SERAPTS, with a view to extending it to the broad spectrum of all creative arts and psychomotor therapies. The SERAPTS was shown to be a valid and sensitive instrument for monitoring the effects of creative arts and psychomotor therapies, indicating healthy self-expression and emotion regulation skills that serve positive self-regulation.

## Introduction

Creative arts and psychomotor therapies are frequently utilised in the treatment of individuals experiencing mental and psychosomatic problems, underpinned by a systematic utilisation of experiential approaches [1,2]. These therapies encompass visual art therapy, dance therapy, drama therapy, music therapy, psychomotor therapy and play therapy. These forms of therapy are particularly prevalent in the treatment of individuals experiencing emotion regulation difficulties or personality disorders [3]. The experiential approach of arts and psychomotor therapies, incorporating music, dance, drama and art, along with attention to body awareness and movement, facilitates the creation of a space for the triggering, exploration and experience of emotions, inner conflicts, the practise of new behaviours, self-reflection and self-insight [4].

The significance and meaningfulness of these forms of arts and psychomotor therapies is recognised by both patients and therapists, despite the paucity of studies indicating their effectiveness [1,4–6]. The scientific evidence for the effectiveness of these therapies in individuals experiencing emotion regulation difficulties or personality disorders is primarily derived from a randomised controlled trial (RCT) [7,8] and two pilot RCTs [9,10]. Arts and psychomotor therapies can be considered for purposes of coming into emotional contact with difficult aspects of patients and their experiences, to work on goals such as regulation of emotions, stress, identity/self-image, self-expression, mood/anxiety, relaxation, changing patterns and social functioning [1]. In addition, there are several other relevant indications in studies that arts and psychomotor therapies are effective in the treatment of emotion regulation problems and personality disorders (e.g., [11–16]). The relevance of these studies is evidenced by the fact that a significant proportion of the study group had been diagnosed with emotion regulation problems or personality disorders.

Notwithstanding these indications, robust scientific evidence regarding the utilisation of arts and psychomotor therapies remains highly sought after [1]. In conducting research, a specific outcome measure is required. As a result, an instrument designed to measure specific aspects of arts and psychomotor therapies is needed. It is important to note that a single instrument that can be utilised across all these forms of therapy is not currently available. Consequently, the outcomes of therapies that are specifically focused on experience must be measured using instruments developed for psychological, often cognitive forms of therapy. This discrepancy may potentially result in a misalignment between the intended and actual outcomes. It is therefore essential to develop a specific outcome measure designed from the principles of arts and psychomotor therapies, in order that specific outcomes can be reliably monitored.

Creative arts and psychomotor therapies constitute components of a comprehensive clinical treatment programme, frequently encompassing a duration of one day. As such, determining the specific and isolated effect of the art therapy intervention itself is challenging [17]. Optimal alignment of treatment can be promoted by regularly evaluating treatment progress with patients [3]. The monitoring of treatment and the discussion of scores with patients has been shown to have a positive impact on the treatment effect [18]. Since the turn of the millennium, there has been a substantial increase in the use of Routine Outcome Monitoring or measurements (ROM) across a range of therapeutic settings [19,20]. ROM is utilised to evaluate treatment outcomes at regular intervals to monitor patients’ progress during treatment and can additionally be employed for effectiveness research [21,22]. ROM is optimally employed as an integral component of therapy, encompassing the decision-making process, treatment progress monitoring, and real-time adjustment when necessary [19,23].

In previous research, the Self-expression and Emotion Regulation in Art Therapy Scale (SERATS) was developed and validated for solely visual art therapy [5,24]. The development of this instrument was necessitated by the absence of a specific measurement instrument to monitor and evaluate art therapy. The SERATS is a tool designed to measure the effect of creative arts on the development of healthy self-expressive and emotion regulation skills, which in turn promote positive self-regulation. The development of the SERATS involved rigorous psychometric analysis, ensuring its reliability and validity as a measurement tool.

The initial round of analyses comprised two independent samples (*n = *335; *n = *34) of patients diagnosed with personality disorders of the Cluster B/C variety, which yielded a unidimensional 9-item scale. The scale demonstrated a high internal consistency (*n = *335; Cronbach’s alpha = .94) and high test-retest reliability over a period of 1–3 weeks (*n = *75; *r = *.96). Furthermore, the SERATS demonstrated sensitivity in monitoring the individual changes of participants in art therapy over a period of 13 weeks (*n = *34; t(27)=3.13, p < .004; effect size d = .60) [24]. Nevertheless, it should be noted that the observed change, as measured by a pretest-post-test design, could not be exclusively attributed to the art therapy sessions. It is important to note that the participants in this particular sample received art therapy as a component of a more extensive treatment programme.

In contrast, in a subsequent study (n = 57), the SERATS was used in a randomised controlled trial with an isolated art therapy intervention of 10 weeks. A significant change over time was identified (F(1,31)=111.55, p = .00) with a substantial effect size (d = 2.03) [25]. In the study by Haeyen et al. [24], a further analysis examined the correlations between the SERATS and other relevant instruments including the AAQ-II (mental flexibility; Acceptance and Action Questionnaire) [26], the MAAS [27] and the OQ45 (general mental functioning, i.e.,; Outcome Questionnaire 45) [28], the SMI (presence of different maladaptive and adaptive mental modes; Schema Mode Inventory) [29], and the MHC-SF (social-, emotional-, and psychological well-being, i.e.,; Mental Health Continuum—short form) [30]. The findings indicated a low correlation (r < .4), suggesting that the SERATS exhibited discriminant validity but lacked convergent validity. Overall, the SERATS demonstrated adequate psychometric properties in these studies; however, it lacked convergent validity and consequently construct validity.

Subsequent research was thus conducted [5] in order to examine the convergent validity of the SERATS correlation with the Emotional Expressivity Scale (EES), the Emotion Regulation Strategies for Artistic Creative Activities Scale (ERS-ACA) and the Healthy-Unhealthy Music Scale (HUMS). The study population comprised 179 individuals diagnosed with a personality disorder, characterised by difficulties in self-regulation and emotion regulation, and a population of 53 healthy students who completed the relevant questionnaires (N = 232). The findings demonstrated that the SERATS exhibited high reliability and convergent validity in relation to the ERS-ACA approach strategies and self-development strategies in both populations, and regarding the HUMS healthy scale in the patient population. The SERATS measures have been found to be strongly related to emotion regulation strategies such as acceptance, reappraisal, discharge and problem solving, as well as to improving a sense of self, including self-identity, increased self-esteem and improved agency, and the healthy side of art making. Participants indicated that the SERATS was relatively easy to complete in comparison to the other questionnaires. In conclusion, the SERATS has been shown to be a valid, useful, context-specific and user-friendly instrument for monitoring the outcomes of art therapy, which is indicative of making art in a healthy way that serves positive emotion regulation and self-development. Nonetheless, it should be noted that the SERATS exclusively delineates patients’ experiences within the domain of visual arts therapy. Consequently, the scale is presently only utilised within the domain of art therapy, with its application in other arts or psychomotor therapies not yet explored.

Following the publication of these results, there was a high demand for the use of the SERATS for monitoring and evaluating other creative arts and psychomotor therapies, in addition to art therapy. The present study aims to investigate the reliability and sensitivity to change of the slightly adapted SERATS for the other forms of creative arts and psychomotor therapies for which this scale has been developed and studied. This scale is now referred to as the Self-expression and Emotion-Regulation in Arts and Psychomotor Therapies Scale (SERAPTS). The confirmation of reliability and sensitivity to change would enable the scale to be used for creative arts and psychomotor therapies in general.

## Materials and methods

The objective of this study was to examine the internal consistency and sensitivity to change of the SERATS for other forms of arts and psychomotor therapy, other than art therapy, which has been studied in previous research. A pre-post design study was performed, given that other aspects of the SERATS had previously been examined. The target sample size was set at a minimum of 20 participants per therapy form (music, dance, drama, and psychomotor) for the purpose of assessing internal consistency, with a further 10 participants per therapy form allocated for the evaluation of sensitivity to change.

### Participants

The present study included participants diagnosed with emotion regulation problems or personality disorders classified as Cluster B or C. Furthermore, they were receiving various creative arts and/or psychomotor therapies (i.e., music, dance, drama, psychomotor) as part of a specialised treatment programme. Participants were excluded from the study for the following reasons: being under the age of 18 years; being unable to complete the questionnaire (e.g., severe psychiatric comorbidity, insufficient knowledge and understanding of the Dutch language); and not having provided informed consent to participate in the study. Excluding severe psychiatric comorbidity minimized major medication changes, and medication largely reflected standard treatment. Diagnostic criteria were relatively heterogeneous within Cluster B and C personality pathology, consistent with routine practice in specialized settings.

### Procedures and ethics

The participants were informed about the study by a participating arts/psychomotor therapist, who provided a detailed explanation. The participants were provided with comprehensible information and consent forms. Both verbally and in the written information, it was emphasised that participation was voluntary, and that withdrawal was possible at any moment without any consequence for the receiving treatment. Participants were requested to provide written informed consent prior to the administration of the initial measurement, following a period of at least one week. The participants were informed that they could contact the researcher or the participating therapist if they had any questions regarding the study. The rationale behind this was to ensure the delivery of optimal care, with the results from the measurements being utilised for the evaluation of the treatment.

The present study was conducted over the period from March 1st 2023 to August 31th 2024. The processing and analysis of the data was conducted in a manner that ensured confidentiality. Furthermore, the consent forms and the data were stored in a secure Research Drive environment at HAN University of Applied Sciences. The Ethical Research Committee of the HAN University of Applied Sciences had previously approved the study (ref. no. ECO 415.12/22). The study is in accordance with the Declaration of Helsinki on Ethical Principles for Medical Research Involving Human Subjects.

### Measures

The Self-expression and Emotion Regulation in Arts Therapies Scale (SERATS) [5,7,24] is a reliable, valid and user-friendly instrument to monitor art therapy. The SERATS measures emotion regulation and self-expression in art therapy. The questionnaire contains nine items, with examples including the statements “I am able to depict my feelings in art therapy” and “Making art is kind of an outlet for me”. Participants are invited to indicate their level of agreement with each statement on a 5-point Likert scale, ranging from “Never true” to “Nearly always true”. Scores on this scale are interpreted as follows: a score of ‘Never true’ indicates poor self-expression and emotion regulation in art therapy, while a score of ‘Nearly always true’ indicates strong self-expression and emotion regulation in art therapy.

The SERATS is a one-factor instrument with a high internal reliability (α = .94) and a high test-retest reliability (r = .96). The SERATS has been shown to be sensitive to measuring therapeutic change over time in the context of art therapy [7,24]. The SERATS demonstrates both discriminant and convergent validity [5,7,24]. In addition, the SERATS appears to be comparatively uncomplicated in terms of completion by patients and students in comparison to similar questionnaires [5]. For the described purposes of this study, the SERATS was modified in that wherever art therapy was mentioned, this was replaced by the words: arts and psychomotor therapies. The adapted version is shown in Table 1.

**Table 1:** The SERAPTS.

**SERAPTS**						
Self-expression and Emotion Regulation in Arts and Psychomotor Therapies Scale						
©Haeyen, Noorthoorn, Joosten, 2025						
Name: *(your data will be processed anonymously)*						
Age: Sex: M/ F/ x*						
Date:						
Treatment setting: Clinic/ Multi-day part-time program/ Outpatient*						
Treatment Form: Group/ Individual/ Both*						
Type of arts or psychomotor therapies you have (more answers possible): Visual art therapy, Dance therapy, Drama therapy, Music therapy or Psychomotor therapy*						
* Delete if not applicable						
*INSTRUCTION*						
*The following statements will to a greater or lesser extent apply to you. Please read the statements carefully and answer them right away by ticking the chosen answer. Do not skip questions.*						
	**Item**	1. Never true	2. Seldom true	3. Some-times true	4. Often true	5. (Almost) always true
1	I get in touch with my feelings through the art(s)/ psychomotor therapy working methods					
2	I am able to depict my feelings in art(s)/ psychomotor therapy					
3	Through the art(s)/ psychomotor therapy working methods, I am able to discover what is at play within me					
4	I am able to express my feelings through the art(s)/ psychomotor therapy working methods					
5	I am able to make things fall into place in art(s)/ psychomotor therapy					
6	Art(s)/ psychomotor therapy is a kind of outlet for me					
7	An art(s)/ psychomotor therapy experience or piece of art I have created can help me hold on to a particular feeling					
8	I apply the new behaviour that I have been experimenting with in art(s)/ psychomotor therapy outside of the therapy setting					
9	I gain greater insight into my psyche through art(s)/ psychomotor therapy					

In this study, the initial measurement was conducted at random during the treatment period. Consequently, the sample comprised a mixture of participants who had recently initiated treatment and those who had been in treatment for a considerable duration. It is imperative to note that the inclusion criteria stipulated that participants must have been undergoing treatment for a minimum of three months (rather than at the conclusion of their treatment). The objective of this study was not to measure the effect of treatment, but rather to ascertain whether the instrument can measure change over time. Participants might have received various forms of art therapy during their treatment but completed the SERAPTS per art therapy form separately. The second measurement was taken three months after the first, as recommended by the SERATS [5]. The rationale behind this recommendation is that the use of the instrument at more frequent intervals could potentially interfere with the therapeutic process, while its less frequent use may be less efficacious. Furthermore, a range of participant characteristics were collected, including age, sex, treatment setting and treatment form.

### Statistical analyses

Participant characteristics were summarised using descriptive statistics. Cronbach’s alphas were conducted for the SERAPTS of arts and psychomotor therapies separately. The sample size was determined at a minimum of 20 participants per therapy form, based on the number of SERAPTS items with an anticipated Cronbach’s alpha of 0.70 and a power of 90% [31]. Inter-item reliability of the SERATS was examined in our previous validation study, which demonstrated good internal consistency (Cronbach’s α values reported in Haeyen et al., [5,24]. For this reason, we did not repeat this analysis in the present study, which focused on validating the questionnaire for multiple therapy modalities beyond art therapy. Paired sample t-tests, with a minimum of 10 participants per therapy form, were used to examine sensitivity to change between first and second measurement 3 months later. This test was two-sided, with a p-value of.05 or less being considered to indicate statistical significance. Finally, Cohen’s d was computed in order to establish the effect size [32]. According to Cohen, effect sizes of 0.2, 0.5, and 0.8 are classified as small, medium, and large, respectively. All statistical analyses were performed using SPSS 27 for Windows [33,34].

## Results

The present study comprised a total of 96 participants. Table 2 presents the demographic details of the participants. The majority of participants were female and received outpatient group treatment.

**Table 2:** Demographics of the participants (N = 96).

Characteristic	*n*	
**Age, mean (SD), range, years**	96	37.0 (11.4), 21-70
**Gender, %**		
Female	62	34.4
Male	33	64.6
X	1	1.0
**Setting, %**		
Inpatient	6	6.4
Day therapy (3 days/week)	63	67.0
Outpatient	25	26.6
**Treatment form, %**		
Group	60	63.2
Individual	20	21.2
Both	15	15.8
**Therapy form, %**		
Dance	20	20.8
Drama	22	22.9
Music	22	22.9
Psychomotor	32	33.3

### Reliability

As illustrated in Table 3, the internal consistency of the complete dataset and each form of therapy is indicated by Cronbach’s alpha. A high level of reliability was demonstrated by SERAPTS across all therapy forms, with a Cronbach’s alpha of.95 being attained. Additionally, the same high reliability scores of 0.95 were observed for music and psychomotor therapy. Furthermore, high reliability scores of 0.88 and 0.89 were found for dance and drama therapy, respectively.

**Table 3:** Internal consistency of the SERAPTS per therapy form.

SERAPTS	*n*	Cronbach’s Alpha
**TOTAL**	93	0.95
**Dance therapy**	20	0.88
**Drama therapy**	22	0.89
**Music therapy**	22	0.95
**Psychomotor therapy**	29	0.95

### Sensitivity to change

A total of 67 participants completed both measurements of the SERAPTS. A significant difference was measured between both measurements (t = −4.39, p < .001). The analysis revealed an increase in SERAPTS scores within a period of three months, indicating an enhancement in emotion regulation and self-expression across all therapeutic modalities. For detailed information per therapy form, please see Table 4. A notable increase in score was observed across all therapy forms. Significant changes were observed within two therapy forms. The objective of these analyses was to ascertain the sensitivity of the SERAPTS in measuring therapeutic change over time. The analysis yielded an overall medium effect size for this variable (d = 0.54). Higher effect sizes were identified for the two therapy forms that demonstrated significant change over time (d = 0.77; d = 0.91). The comparatively lower effect sizes observed in the Dance Therapy and Music Therapy groups can be attributed to the too small sample sizes in these groups, clearly below 20 cases, the minimum case number to achieve a power of 0.8 and an alpha of 0.05 (34).

**Table 4:** Sensitivity to change (3 months) of the SERAPTS.

SERAPTS mean (SD)	*n*	T1	T2	t-value, p-value	ES (d)
**TOTAL**	67	28.78 (8.08)	31.01 (7.69)	−4.39, < 0.001**	0.54
**Dance therapy**	13	36.46 (5.64)	37.00 (5.12)	−0.86; 0.41	0.24
**Drama therapy**	19	29.90 (5.29)	32.79 (3.92)	−3.33; 0.004**	0.77
**Music therapy**	14	25.86 (7.57)	26.64 (9.72)	−0.54; 0.60	0.14
**Psychomotor therapy**	21	24.95 (8.56)	28.62 (7.63)	−4.17; < 0.001**	0.91

## Discussion

The objective of the present study was to evaluate the reliability and sensitivity to change of the Self-expression and Emotion-Regulation in Arts and Psychomotor Therapies Scale (SERAPTS) in relation to dance, drama, music and psychomotor therapy. The results of this study demonstrated analogous findings to those of previous studies and indicated that the SERAPTS exhibited high to excellent reliability. Furthermore, a significant medium effect size was observed for the sensitivity of change over a three-month period.

A divergence in the significance and effect sizes of the therapy forms was observed. This discrepancy can be attributed to the small number of participants within some of the therapy groups consequently not demonstrating significant change over time. Overall effect size was above the threshold for moderate, which is given the number of cases quite satisfactory. Consequently, the SERAPTS has been determined to be a reliable and sensitive instrument for monitoring the effects of art, dance, drama, music, and psychomotor therapy. Previous research has already established the validity of the scale [5,24]. Monitoring is especially useful as an effect measurement during treatment and to adjust ongoing treatments if necessary, when the client is not “on track”. To this end, the discussion of the scores with the patient is of paramount importance. The subsequent discussion is imperative for the effective implementation of the scale.

The provision of feedback regarding routine outcome monitoring scores has some potential to engender adverse effects in cases where such feedback is repeatedly received by vulnerable patients suffering from severe psychopathology or personality disorder [35]. This is one of the factors that should be taken into account when selecting a monitoring instrument. The SERAPTS has been developed to measure positive outcomes in an accessible manner, ensuring ease of completion and brevity.

A limitation of the SERAPTS is that the questionnaire is context specific. Consequently, its utilisation is contingent upon the participant having undergone one or more sessions (1–3 sessions) of arts and psychomotor therapy sessions. The initial measurement is only feasible after at least one session. Conversely, the context-specificity of the SERAPTS is also a strength, in that it enables the focus to be directed specifically on this form of therapy within a broader treatment programme consisting of several components. This approach aligns well with the prevailing circumstances in clinical practice, where arts and psychomotor therapies frequently constitute components of an integrated treatment programme.

It is also important to emphasise that the SERAPTS specifically focuses on self-expression and emotion regulation, whereas creative arts and psychomotor therapies consist of more aspects. This focus was deliberately chosen because these are important core objectives of creative arts and psychomotor therapies, as for example with clients with personality disorders. It is acknowledged that other domains of creative arts and psychomotor therapies, such as structuring skills, material handling, experimentation with varied behavioural expressions, and the therapeutic alliance, are not encompassed in the present questionnaire. Emotion regulation is not a strictly delimited construct. In line with current perspectives of Gross, Neumann and colleagues [e.g., 36, 37], we conceptualize emotion regulation as a multidimensional process, encompassing awareness, acceptance, modulation, and expression of emotions. Within personality disorders, dysregulation manifests across this continuum rather than as a discrete, uniform deficit. The SERAPTS aims to capture this broader process-based understanding by assessing observable change in emotion awareness, tolerance, and expression through artmaking, domains empirically linked to emotion regulation and dysregulation. Thus, the instrument’s operationalization is complementary to, rather than divergent from, clinically recognized mechanisms of emotion regulation. Self-expression and emotion regulation are assessed as core domains in the SERAPTS because creative arts and psychomotor therapies engage clients in expressive and bodily activities that elicit, modulate, and integrate their self-expression and emotional experiences, making changes in emotion regulation a key indicator of therapeutic progression.

At the present time, no norming is available for the SERAPTS. The establishment of such a norm has proven challenging, primarily due to the context-specific nature of the questionnaire. Consequently, the establishment of a healthy norm group, against which to benchmark, is complicated by the absence of a creative arts and psychomotor therapies component in such a group. However, future research plans may include norm development or cross-cultural validation. Different translations have been established based on international interest in this questionnaire. A norm group would strengthen the generalizability of the results. The norm puts individual scores into perspective and makes them interpretable, comparable, and clinically or practically useful. In any case, with this study we have shown how the SERAPTS appears to improve the measurability of art and psychomotor therapies.

In conclusion, the SERAPTS has been demonstrated to be an internally consistent, reliable, valid and sensitive instrument for monitoring the effect of the broad spectrum of creative arts and psychomotor therapies. The SERAPTS indicates healthy self-expression and emotion regulation skills that serve positive self-regulation. It is imperative to acknowledge that healthy self-expression and emotion regulation are fundamental basic needs that contribute to mental health and well-being. The questionnaire has been developed for the purpose of evaluating progress in creative arts and psychomotor therapy and facilitating research on the effects of these interventions.

## References

[1] Haeyen S. Effects of arts and psychomotor therapies in personality disorders: developing a treatment guideline based on a systematic review using GRADE. Front Psychiatry. 2022;13:878866. 35782411 10.3389/fpsyt.2022.878866 PMC9243752

[2] Borgesius E, Visser ECM. [Arts therapy and day care in the medical mental healthcare]. Diemen (Netherlands): Zorginstituut Nederland; 2015. Dutch. https://www.zorginstituutnederland.nl/binaries/zinl/documenten/standpunten/2015/10/29/standpunt-vaktherapie-en-dagbesteding-in-de-geneeskundige-ggz/Vaktherapie+en+dagbesteding+in+de+geneeskundige+GGZ.pdf

[3] Haeyen S. Schema focused working methods for arts and body-based therapies: a practical guide. New York: Routledge. 2024.

[4] Haeyen S, Chakhssi F, van Hooren S. Benefits of art therapy in people diagnosed with personality disorders: a quantitative survey. Front Psychology. 2020;11:686. 32351431 10.3389/fpsyg.2020.00686 PMC7174707

[5] Haeyen S, Noorthoorn E. Validity of the Self-Expression and Emotion Regulation in Art Therapy Scale (SERATS). PLoS One. 2021;16(3):e0248315. doi: 10.1371/journal.pone.0248315 33690731 PMC7946186

[6] Solli HP, Rolvsjord R, Borg M. Toward Understanding Music Therapy as a Recovery-Oriented Practice within Mental Health Care: A Meta-Synthesis of Service Users’ Experiences. J Music Ther. 2013;50(4):244–73. doi: 10.1093/jmt/50.4.244 25014667

[7] Haeyen S, van Hooren S, van der Veld W, Hutschemaekers G. Efficacy of Art Therapy in Individuals With Personality Disorders Cluster B/C: A Randomized Controlled Trial. J Pers Disord. 2018;32(4):527–42. doi: 10.1521/pedi_2017_31_312 28926306

[8] Haeyen S, van Hooren S, van der Veld WM, Hutschemaekers G. Promoting mental health versus reducing mental illness in art therapy with patients with personality disorders: A quantitative study. The Arts in Psychotherapy. 2018;58:11–6. doi: 10.1016/j.aip.2017.12.009

[9] van den Broek E, Keulen-de Vos M, Bernstein DP. Arts therapies and Schema Focused therapy: A pilot study. The Arts in Psychotherapy. 2011;38(5):325–32. doi: 10.1016/j.aip.2011.09.005

[10] Keulen-de Vos M, van den Broek EPA, Bernstein DP, Vallentin R, Arntz A. Evoking emotional states in personality disordered offenders: An experimental pilot study of experiential drama therapy techniques. The Arts in Psychotherapy. 2017;53:80–8. doi: 10.1016/j.aip.2017.01.003

[11] Karterud S, Pedersen G. Short-term day hospital treatment for personality disorders: benefits of the therapeutic components. Ther Communities. 2004;25(1):43–54.

[12] Kipper DA, Ritchie TD. The effectiveness of psychodramatic techniques: A meta-analysis. Group Dynamics: Theory, Research, and Practice. 2003;7(1):13–25. doi: 10.1037/1089-2699.7.1.13

[13] Gold C, Solli HP, Krüger V, Lie SA. Dose-response relationship in music therapy for people with serious mental disorders: systematic review and meta-analysis. Clin Psychol Rev. 2009;29(3):193–207. doi: 10.1016/j.cpr.2009.01.001 19269725

[14] Gebhardt S, Kunkel M, Von Georgi R. Emotion modulation in psychiatric patients through music. Music Percept. 2024;31(5):485–93.

[15] Leirvåg H, Pedersen G, Karterud S. Long-term continuation treatment after short-term day treatment of female patients with severe personality disorders: Body awareness group therapy versus psychodynamic group therapy. Nord J Psychiatry. 2010;64(2):115–22. doi: 10.3109/08039480903487525 20392134

[16] Zwets AJ, Hornsveld RHJ, Muris P, Kanters T, Langstraat E, van Marle HJC. Psychomotor Therapy as an Additive Intervention for Violent Forensic Psychiatric Inpatients: A Pilot Study. International Journal of Forensic Mental Health. 2016;15(3):222–34. doi: 10.1080/14999013.2016.1152613

[17] Bateman AW, Fonagy P. Mentalization-based treatment of BPD. J Pers Disord. 2004;18(1):36–51. doi: 10.1521/pedi.18.1.36.32772 15061343

[18] de Jong K, Douglas S, Wolpert M, Delgadillo J, Aas B, Bovendeerd B, et al. Using Progress Feedback to Enhance Treatment Outcomes: A Narrative Review. Adm Policy Ment Health. 2025;52(1):210–22. doi: 10.1007/s10488-024-01381-3 38733413 PMC11703940

[19] Barkham M, De Jong K, Delgadillo J, Lutz W. Routine Outcome Monitoring (ROM) and Feedback: Research Review and Recommendations. Psychother Res. 2023;33(7):841–55. doi: 10.1080/10503307.2023.2181114 36931228

[20] de Jong K, Conijn JM, Gallagher RAV, Reshetnikova AS, Heij M, Lutz MC. Using progress feedback to improve outcomes and reduce drop-out, treatment duration, and deterioration: A multilevel meta-analysis. Clin Psychol Rev. 2021;85:102002. doi: 10.1016/j.cpr.2021.102002 33721605

[21] de Beurs E, den Hollander-Gijsman ME, van Rood YR, van der Wee NJA, Giltay EJ, van Noorden MS, et al. Routine outcome monitoring in the Netherlands: practical experiences with a web-based strategy for the assessment of treatment outcome in clinical practice. Clin Psychol Psychother. 2011;18(1):1–12. doi: 10.1002/cpp.696 20238371

[22] Van Os J. Van routine outcome naar routine omgeving monitoring: Een nieuwe methode om verzekeraars en ggz te helpen met focus en doelmatigheid. Utrecht: UMC Utrecht. 2020.

[23] Lambert MJ, Whipple JL, Kleinstäuber M. Collecting and delivering progress feedback: A meta-analysis of routine outcome monitoring. Psychotherapy (Chic). 2018;55(4):520–37. doi: 10.1037/pst0000167 30335463

[24] Haeyen S, van Hooren S, van der Veld WM, Hutschemaekers G. Measuring the contribution of art therapy in multidisciplinary treatment of personality disorders: The construction of the Self-expression and Emotion Regulation in Art Therapy Scale (SERATS). Personal Ment Health. 2018;12(1):3–14. doi: 10.1002/pmh.1379 28730717 PMC5836990

[25] Haeyen S. Effects of art therapy: the case of personality disorders cluster B/C. Nijmegen (Netherlands): Radboud University. 2018.

[26] Jacobs N, Kleen M, de Groot F, A-Tjak J. Measuring experiential avoidance: the Dutch version of the Acceptance and Action Questionnaire-II (AAQII). Gedragstherapie. 2008;41(4):349–61.

[27] Schroevers M, Nyklıcek I, Topman R. Validation of the Dutch version of the Mindful Attention Awareness Scale (MAAS). Gedragstherapie. 2008;41(3):225–40.

[28] Lambert MJ, Burlingame GM, Umphress V, Hansen NB, Vermeersch DA, Clouse GC. The reliability and validity of the outcome questionnaire. Clin Psychol Psychother. 1996;3(4):249–58.

[29] Young J, Arntz A, Atkinson T, Lobbestael J, Weishaar M, van Vreeswijk F, et al. The Schema Mode Inventory (SMI). Version 1. New York: Schema Therapy Institute; 2007.

[30] Lamers SMA, Westerhof GJ, Bohlmeijer ET, ten Klooster PM, Keyes CLM. Evaluating the psychometric properties of the Mental Health Continuum-Short Form (MHC-SF). J Clin Psychol. 2011;67(1):99–110. doi: 10.1002/jclp.20741 20973032

[31] Bujang MA, Omar ED, Baharum NA. A Review on Sample Size Determination for Cronbach’s Alpha Test: A Simple Guide for Researchers. Malays J Med Sci. 2018;25(6):85–99. doi: 10.21315/mjms2018.25.6.9 30914882 PMC6422571

[32] Cohen J. Statistical power analysis for the behavioural sciences. 2nd ed. New York: Lawrence Earlbaum Associates; 1988.

[33] IBM Corp. IBM SPSS statistics for Windows. Armonk (NY): IBM Corp. 2020.

[34] Mouelhi Y, Jouve E, Castelli C, Gentile S. How is the minimal clinically important difference established in health-related quality of life instruments? Review of anchors and methods. Health Qual Life Outcomes. 2020;18(1):136. doi: 10.1186/s12955-020-01344-w 32398083 PMC7218583

[35] de Jong K, Segaar J, Ingenhoven T, van Busschbach J, Timman R. Adverse Effects of Outcome Monitoring Feedback in Patients With Personality Disorders: A Randomized Controlled Trial in Day Treatment and Inpatient Settings. J Pers Disord. 2018;32(3):393–413. doi: 10.1521/pedi_2017_31_297 28594629

[36] Gross JJ. Emotion Regulation: Current Status and Future Prospects. Psychological Inquiry. 2015;26(1):1–26. doi: 10.1080/1047840x.2014.940781

[37] Neumann A, van Lier PAC, Gratz KL, Koot HM. Multidimensional assessment of emotion regulation difficulties in adolescents using the Difficulties in Emotion Regulation Scale. Assessment. 2010;17(1):138–49. doi: 10.1177/1073191109349579 19915198

